# Factors associated with the risk perception and purchase decisions of Fukushima-related food in South Korea

**DOI:** 10.1371/journal.pone.0187655

**Published:** 2017-11-08

**Authors:** Dalnim Lee, Songwon Seo, Min Kyoung Song, Hyang Ki Lee, Sunhoo Park, Young Woo Jin

**Affiliations:** 1 National Radiation Emergency Medical Center, Korea Institute of Radiological and Medical Sciences, Seoul, Republic of Korea; 2 Consumers Union of Korea, Seoul, Republic of Korea; University of South Carolina, UNITED STATES

## Abstract

Following the Fukushima nuclear power plant accident, the risk level perceived by Koreans on the radioactive contamination of Japanese food that is being distributed in Korea remains high. Many of these perceptions are based on subjective risk perception rather than an objective measure with scientific evidence, which makes communicating risks more difficult; therefore, it is critical to understand factors associated with risk perception for effective risk communication. In this study, we identified variables that are associated with buying tendencies and opinions about the regulatory policy of Japanese seafood after the accident. A survey was conducted with 1045 adults aged over 20 years in Korea. The majority (68.8%) responded that they would not purchase Japanese seafood when radioactivity levels in the food were non-detectable. Moreover, 82.2% responded that the current levels of import restrictions on Japanese seafood must be maintained. Despite many concerns regarding the exposure to radiation and the effects from food products following the Fukushima accident, the opportunities to encounter and to collect correct information remain limited and average radioactive knowledge scores were low (3.63 out of 9). Of the various characteristics associated with purchase decisions and agreement on the current import restraints of Japanese seafood, trust levels in the government and the mass media for providing information on radioactivity were major factors that influenced risk perception. While the scope of this study was limited to seafood, it is very closely tied to daily lives, where we revealed differences about risk perceptions and agreement on import restraints of Japanese seafood per a complex mixture of individual characteristics and the surrounding environment. These results provide useful information to understand the risk perception of the potential radioactive contamination of food and to predict the public’s responses to food consumption and import restraint policies due to nuclear accidents in neighboring countries.

## Introduction

Following the Fukushima-Daiichi nuclear plant accident that occurred in March of 2011 in Japan, the world responded with concerns towards the radioactivity contamination of Japanese food and a subsequent decrease in the purchase of Japanese food [1–3]. Where the accident had occurred, the in-food levels of radioactive cesium were strengthened to 100 Bq/kg (April 2012) by the government of Japan, which was followed by the Korean government’s mirroring of these regulations for Japanese food products (including dairy products) in Korea, strengthening general food products from 370 Bq/kg to 100 Bq/kg, and milk and baby’s food products (including baby formula) from 370 Bq/kg to 50 Bq/kg. While the Korean government consistently requested information on the management of radioactive contamination of Japanese food and its transparency, the Japanese media reported daily discharges of hundreds of tons of radioactive contaminated material. This led the Korean government to introduce temporary special measures on Japanese food, and a strengthening of radioactive cesium limits on all imported and domestically distributed food products to 100 Bq/kg (tentative levels, and for Japanese-imported potable water, at 10 Bq/kg, milk and dairy products at 50 Bq/kg) [4]. In addition, the Korean government banned all imports of seafood from the Fukushima Prefecture and 8 neighboring Prefectures of Aomori, Iwate, Miyagi, Ibaraki, Chiba, Gunma, and Tochigi, and began to require additional reports on other radioactive nuclides including strontium and plutonium, even though only a minimal level of radioactive cesium was found in Japanese-imported seafood from other regions. In May 2015, Japan filed a case to the World Trade Organization (WTO) stating that Korea placed excessive regulations on their products compared to other countries [5,6]. However, some organizations in Korea asked for a complete import ban from Japanese food products [7,8], and following the news releases [9,10] on the discharge of contaminated water and the presence of radioactive material in seafood, the majority of Korean citizens remained concerned about the radioactive contamination of Japanese seafood and the related government management policies. As such, the need for effective risk communication by the government on radioactive contamination is required. To achieve this, one must first identify the level of risk perception on radioactive contaminated food and the factors influencing such perceptions.

Most previous studies on risk perception towards food have focused on the risks inherent to food such as calories and ingredients; on the risks of food processing [5,11,12]; or on the management of food such as bacteria, pesticides, and food poisoning [13,14]. While there were a few studies on the perception of Japanese food following the Fukushima accident, most studies examined Japanese citizens [9,15–17]. There were also a few studies on American consumers [3] and Korean consumers [18] on the perception of seafood after the Fukushima accident; however, they mainly focused on the general level of risk perception and awareness about food safety issues related to radioactive contaminations. Furthermore, those studies had limitations in understanding and confirming factors relating to public opinion on regulatory policy regarding potential radioactive contamination of food. Therefore, this study investigated factors associated with seafood-purchasing behavior after the Fukushima accident and the regulatory policy opinions about the potential radioactive contamination of food to understand risk perception differences among the general population, thereby encouraging communication strategies for radiation-related food safety.

## Methods

### Survey design

Overall, 1050 adults aged 20 to 69 years from across Korea except for Jeju province were invited to participate. Stratified random sampling was used considering the population distribution factors such as region and sex, and the survey was designed with a sampling error of ±3% at a 95% confidence interval. The survey was conducted through face-to-face interviews from November 7 to 17, 2014 in places with a large floating population such as a market and a park. The survey questions were developed by experts in the field of radiation, food safety, risk perception, and communication, and the final copy of the survey questions was reviewed by a committee of civilian experts, which consisted of 10 experts from academia, non-governmental organizations (NGO), and public office, to clarify and validate the questionnaire, and to amend the questionnaire with more easily understandable terms, especially for questions examining radiation knowledge and the contents of basic information about radioactivity and radiation. The survey was composed of 29 main questions: 6 questions on the perception of radioactive material, 7 questions on food purchase, 1 question on knowledge about radioactivity and radiation (9 sub-questions for the knowledge score), 9 questions on the confidence level of radioactive material management and perceptions on safety management policies, and 6 questions addressing the participants’ characteristics including sex, age, region, and education level. The questionnaire mainly focused on seafood; the survey was originally planned to understand consumers’ perceptions of seafood following the implementation of temporary special measures regarding Japanese seafood to protect against leakage of contaminated water from a storage tank at Japan’s crippled Fukushima nuclear power plant. The questionnaire is available in both Korean (original language; S1 Appendix) and English (S2 Appendix). This study was approved by the institutional review board at the Korea Institute of Radiological & Medical Sciences (IRB number: K-1608-002-047).

### Statistical analysis

The categorical questions were summarized with percentages, and the knowledge levels for radioactivity were scored by adding the correct answers to the 9 questions (range: 0–9). To identify variables associated with the three main questions (i.e., change of seafood purchase frequency after the Fukushima accident, purchase intentions for Japanese seafood with non-detectable levels of radiation, and agreement on the maintenance of current levels of import restraints on Japanese seafood), each question was set as the outcome variable, and univariate and multivariate regression analyses were conducted with backward selection (p < 0.2 for variable entry, p < 0.05 for variable to stay). A logistic analysis was used to identify factors associated with decreases in seafood purchase frequency and purchase intentions of Japanese seafood with non-detectable levels of radiation. The level of agreement on the maintenance of current levels of import restraints on Japanese seafood was measured using a 5-point Likert scale and analyzed using a linear regression analysis to identify its related factors. Statistical significance was defined as a two-tailed p-value < 0.05 and no multiplicity adjustment was made.

## Results

### Participants’ characteristics and survey responses

Of 1050 people originally contacted, 1045 responded to the survey (response rate = 99.5%; 502 males, 543 females). Participants’ demographic characteristics are provided in Table 1.

**Table 1:** Participants’ characteristics.

	Frequency	%
Sex		
Male	502	48.0
Female	543	52.0
Age		
20s	187	17.9
30s	238	22.8
40s	254	24.3
50s	244	23.4
60s or above	122	11.7
Education		
High school or below	280	26.8
Post-secondary graduate or above	764	73.1
No response	1	0.1
Occupation		
Homemaker	189	18.1
Office worker*	472	45.2
Student	105	10.1
Other	279	26.7
Age of family members		
Elementary school student or younger	212	20.3
Middle or high school student	239	22.9
Adult	409	39.1
None	185	17.7

*Office worker included professional and public official.

Participants’ channels of acquiring information about radiation are provided in Table 2. Of the respondents, 47.9% listed TV as their preferred source of information; 95.1% of respondents related the word “radioactivity” with “nuclear weapons,” “nuclear plant accident,” or “Fukushima nuclear power plant accident.” The reason the respondents felt that their health was threatened was because “radiation was used around us whether we know it or not” (38.4%) and because “radiation is continuously released by the Fukushima nuclear power plant or nuclear power plants in South Korea” (35.4%). Participants’ seafood purchasing tendencies are presented in Table 3. The average radiation knowledge score for all respondents is presented in Table 4. Participants showed more confidence in the Korean government compared to the Japanese government; however, participants typically had a low level of confidence in both governments. The majority were concerned about news bulletins reporting on the increase in discovery of small amounts of radioactive material, and responded that the current levels of import restraints for Japanese seafood should be maintained despite trade clashes with Japan; 42.7% responded in favor of a complete trade ban until the Fukushima nuclear power plant radioactivity issue was resolved; and 40.8% responded that bans on products from specific regions or on specific items should be implemented (Table 4).

**Table 2:** Participants’ channels of information acquisition and perceptions on radiation.

Items	Response category	Frequency (%)
Experience receiving information on radiation	Yes	399 (38.2)
	No	646 (61.8)
Key information provider (When the respondent had received information)	Mass media	237 (59.4)
	Speeches and training	71 (17.8)
	Social-networking sites or friends	63 (15.8)
	Government or public institutions	12 (3.0)
	Other	16 (4.0)
Association with radioactivity	Hospitals	33 (3.2)
	Nuclear weapons and nuclear power plants	510 (48.8)
	Fukushima nuclear power plant accident	484 (46.3)
	Radon	8 (0.8)
	Other	6 (0.6)
	No response	4 (0.4)
Concerns with the health implications from radiation exposure	Yes	477 (45.6)
	No	264 (25.3)
	Do not care	304 (29.1)

**Table 3:** Perception of information provisions and seafood purchase tendencies.

Items	Response category	Frequency (%)
Foods that should have their origins checked	All products	613 (58.7)
	Baby food	50 (4.8)
	Seafood	282 (27.0)
	Rice	56 (5.4)
	Specific products	44 (4.2)
Seafood purchase tendencies	Only of Korean origin	440 (42.1)
	All seafood despite origin	290 (27.8)
	Avoid seafood from Japan only	268 (25.6)
	Do not purchase seafood regardless of origin	20 (1.9)
	Do not purchase as determining origin is difficult	27 (2.6)
Seafood purchase frequencies after the Fukushima nuclear power plant accident	Increased	6 (0.6)
	No change	366 (35.0)
	Decreased	614 (58.8)
	Do not purchase	59 (5.7)
Purchase intention for Japanese seafood when radiation is non-detectable level	Purchase	108 (10.3)
	Do not know	218 (20.9)
	Do not purchase	719 (68.8)
Awareness that the MFDS* updates information on radioactivity in imported foods on its website daily	Yes	182 (17.4)
	No	863 (82.6)
Utilization of information from MFDS*	Yes	185 (17.7)
	No	860 (82.3)

* MFDS: Ministry of Food and Drug Safety.

**Table 4:** Radiation knowledge and confidence in the governments’ food management.

Items	Response category	Frequency (%)
Knowledge levels of radioactivity	Average (± Standard Deviation)	3.63 (± 2.07)
Confidence in the information provided by the Japanese government	Not at all confident	266 (25.5)
	Not confident	419 (40.1)
	Average	277 (26.5)
	Confident	77 (7.4)
	Very confident	6 (0.6)
Confidence in the information provided by the Korean government	Not at all confident	119 (11.4)
	Not confident	326 (31.2)
	Average	462 (44.2)
	Confident	127 (12.2)
	Very confident	11 (1.1)
Concerns with news bulletins	Not concerned at all	6 (0.6)
	Not concerned	20 (1.9)
	Do not care	137 (13.1)
	Concerned	622 (59.5)
	Very concerned	260 (24.9)
Confidence in the information provided by the mass media	Not at all confident	37 (3.5)
	Not confident	173 (16.6)
	Average	387 (37.0)
	Confident	430 (41.1)
	Very confident	18 (1.7)
Confidence in the management of radioactivity in food	Very incompetent	150 (14.4)
	Incompetent	372 (35.6)
	Average	466 (44.6)
	Good	51 (4.9)
	Very good	6 (0.6)
Agreement with maintaining the current level of import restraints of Japanese seafood even if there is trade friction with Japan	Not at all	24 (2.3)
	No	36 (3.4)
	Average	81 (7.8)
	Yes	493 (47.2)
	Definitely yes	366 (35.0)
	Not sure	45 (4.3)
Restraints measures (only responders who agree with import restraints of Japanese seafood)	Must ban all imports of Japanese food products until the problem of radiation from the Fukushima nuclear power plant is fully resolved	365 (42.7)
	Must ban all imports from specific regions (prefectures) or on specific items (e.g., seafood) for the time being	348 (40.8)
	Decide depending on radiation tests, but lower the allowed amounts of radiation	123 (14.4)
	Limit only those in excess of allowed amounts and carry on radiation tests	18 (2.1)

### Factors associated with purchase decisions and agreement with current import restraints of Japanese seafood

#### Changes in the purchase frequency of seafood following the Fukushima nuclear power plant accident

**Fig 1:**
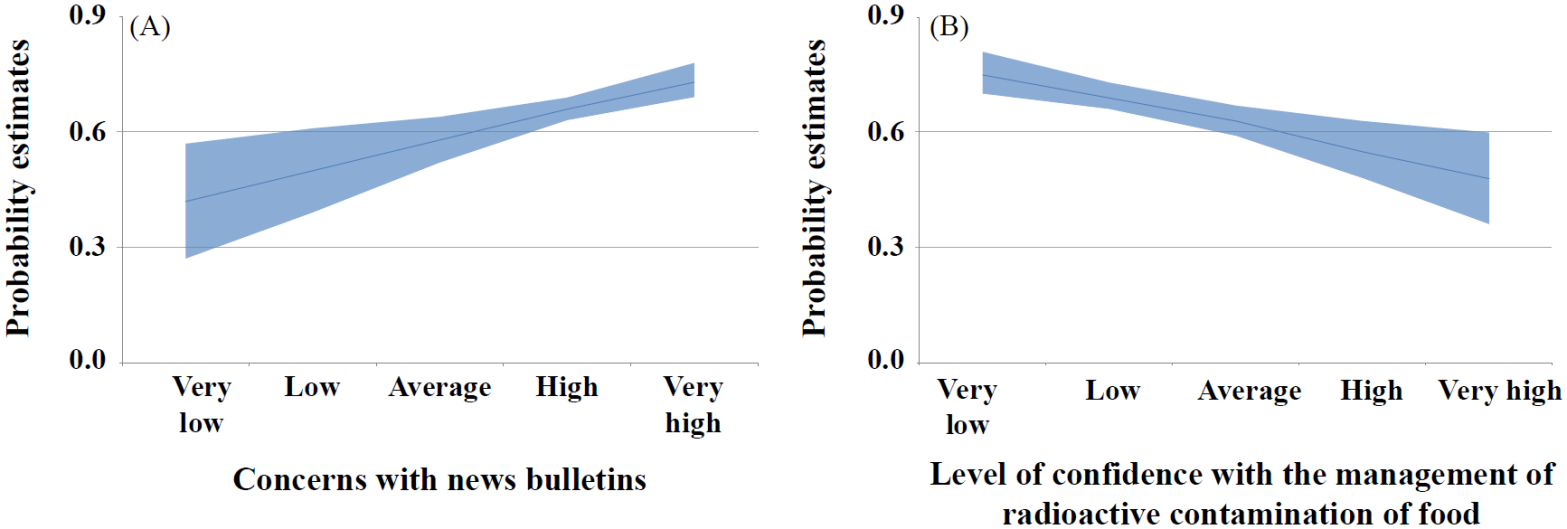
Changes in seafood purchase frequencies after the Fukushima accident per concerns with news bulletins and the level of confidence in the management of radioactive contamination of food. The x-axis denotes the concerns with news bulletins (A) and the level of confidence in the management of radioactive contamination of food (B). The y-axis denotes probability estimates with the decrease in seafood purchase frequencies after adjusting for significant variables in the multivariate model. The 95% confidence interval on the estimates is shown in blue shading. Higher concerns with news bulletins increased the probability of not purchasing seafood (A), and the higher the trust in management of radioactive contamination of food, the lower the probability of non-purchases (B).

The majority (64.4%) of respondents answered that the frequency of their seafood purchases “had decreased or they did not purchase altogether” after the Fukushima nuclear power plant accident. To identify variables associated with the response, a logistic regression analysis was conducted. In the univariate analysis, we identified significant factors related to the decrease in purchasing seafood including information experience, concerns with the health effects of radiation exposure, the source of radioactive material included in agricultural products (e.g., nuclear power plants), important factors considered when purchasing food (e.g., origin, safety, and price), types of food that have their origins checked (e.g., seafood), understanding and utilizing information provided by the Ministry of Food and Drug Safety (MFDS), low confidence level in the government and mass media when providing radioactive information, low confidence in the management of radioactive contamination of food, concerns with news bulletins, and living with a child or adolescent (Table 5). While an increase in seafood purchases was found in respondents with higher education levels, both age and sex were not related to the frequency of purchasing seafood. The multivariate analysis indicated that concerns with the health effects of radiation exposure, the source of radioactive material included in agricultural products, types of food that have their origins checked, awareness and utilization of information provided by the MFDS, concerns with news bulletins, and low confidence in the management of radioactive contamination of food and education levels remained significant (Table 5). Predicted probabilities of the decrease in seafood purchase frequency were high when there were high concerns with news bulletins and low confidence in the management of radioactive contamination of food (Fig 1).

**Table 5:** Factors associated with the decrease in seafood purchase frequencies after the Fukushima nuclear power plant accident.

Variables^a^	Univariate		Multivariate	
	Odds Ratio (95% CI)	*p*-value	Odds Ratio (95% CI)	*p*-value
Received information				
Yes	1.31 (1.01, 1.70)	0.046	-	
Association with radioactivity		0.125		
Nuclear weapons and nuclear power plants	0.78 (0.37, 1.65)	0.517	-	
Fukushima nuclear power plant accident	1.03 (0.49, 2.18)	0.935	-	
Radon	3.5 (0.38, 32.12)	0.268	-	
Other	0.5 (0.09, 2.90)	0.439	-	
Health implications when exposed		0.906		
Child deformity	0.95 (0.71, 1.27)	0.740	-	
Genetic diseases	1.16 (0.76, 1.78)	0.491	-	
Early death due to mysterious disease	0.99 (0.61, 1.6)	0.958	-	
Other	1.2 (0.45, 3.23)	0.717	-	
Concerned with health implications from radiation exposure		< 0.001		< 0.001
Yes	2.32 (1.68, 3.21)	< 0.001	1.82 (1.29, 2.58)	0.001
Do not care	0.7 (0.50, 0.98)	0.038	0.61 (0.43, 0.87)	0.006
Source of radioactive material included in agricultural products		< 0.001		0.006
There cannot be any radioactive material	1.13 (0.53, 2.40)	0.761	1.43 (0.63, 3.23)	0.396
Radioactive material from nuclear power plants (Fukushima and Korea)	2.03 (1.51, 2.72)	< 0.001	1.84 (1.34, 2.53)	< 0.001
Radioactive material residues from nuclear weapon tests in the past	1.41 (0.58, 3.40)	0.448	1.06 (0.41, 2.72)	0.910
Do not know	1.16 (0.81, 1.66)	0.428	1.35 (0.91, 2.00)	0.134
Important factors when purchasing food		< 0.001		
Freshness and expiry date	1.23 (0.85, 1.77)	0.265	-	
Origin	2.62 (1.64, 4.21)	< 0.001	-	
Safety	1.52 (0.99, 2.32)	0.054	-	
Price	0.44 (0.19, 0.97)	0.043	-	
Do not know as no purchase made	3.26 (0.90, 11.89)	0.073	-	
Foods that should have origins checked		0.039		0.024
Baby food	1.39 (0.74, 2.60)	0.301	1.63 (0.84, 3.18)	0.150
Seafood	1.43 (1.06, 1.94)	0.021	1.65 (1.19, 2.29)	0.003
Rice	0.74 (0.43, 1.28)	0.284	0.94 (0.52, 1.71)	0.850
Specific products	0.72 (0.39, 1.32)	0.287	0.90 (0.46, 1.76)	0.760
Awareness of updated information on the MFDS’s website				
Yes	1.52 (1.07, 2.16)	0.019	-	
Utilization of information from MFDS				
Yes	2.05 (1.42, 2.96)	< 0.001	1.94 (1.31, 2.88)	0.001
Radiation knowledge score	0.98 (0.92, 1.04)	0.572	-	
Confidence in the information provided by the Japanese government	0.75 (0.65, 0.87)	< 0.001	-	
Confidence in the information provided by the Korean government	0.84 (0.73, 0.97)	0.014	-	
Concerns with news bulletins	1.64 (1.36, 1.97)	< 0.001	1.4 (1.15, 1.71)	0.001
Confidence in the information provided by the mass media	0.84 (0.73, 0.98)	0.025	-	
Confidence in the management of radioactive contamination of food	0.74 (0.63, 0.86)	< 0.001	0.74 (0.62, 0.88)	0.001
Sex				
Female	0.97 (0.75, 1.25)	0.826	-	
Age		0.149		
30s	0.96 (0.65, 1.42)	0.835	-	
40s	1.44 (0.97, 2.14)	0.073	-	
50s	1.26 (0.85, 1.87)	0.254	-	
60s and older	0.97 (0.6, 1.54)	0.882	-	
Education level				
Post-secondary graduate	0.59 (0.44, 0.8)	0.001	0.56 (0.40, 0.77)	< 0.001
Occupation		0.477		
Professional	0.76 (0.53, 1.09)	0.133	-	
Student	0.78 (0.47, 1.29)	0.335	-	
Self-employed and other	0.77 (0.52, 1.14)	0.189	-	
Age of family members		0.022		
Elementary school and younger	1.73 (1.14, 2.62)	0.010	-	
Middle or high school students	1.47 (0.98, 2.19)	0.060	-	
Adults (over 20 years)	1.11 (0.78, 1.58)	0.571	-	
Number of observations			1044	
Hosmer-Lemeshow goodness of fit			0.261	

^a^Reference categories for each independent variable: experience of information receipt (no), association with radioactivity (hospitals), health influence when exposed to radiation exposure (cancer), health influence concerns from radiation (no), type of radiation included in agricultural products (natural radiation), important factors in food purchase (quality and taste), products whose origin is checked (all products), awareness of updated information on the MFDS’s website (No), utilization of information from MFDS (no), sex (male), age (20s), education levels (high school or below), occupation (housewives), age of family members (none).

#### Intentions to purchase Japanese seafood with non-detectable levels of radiation

**Fig 2:**
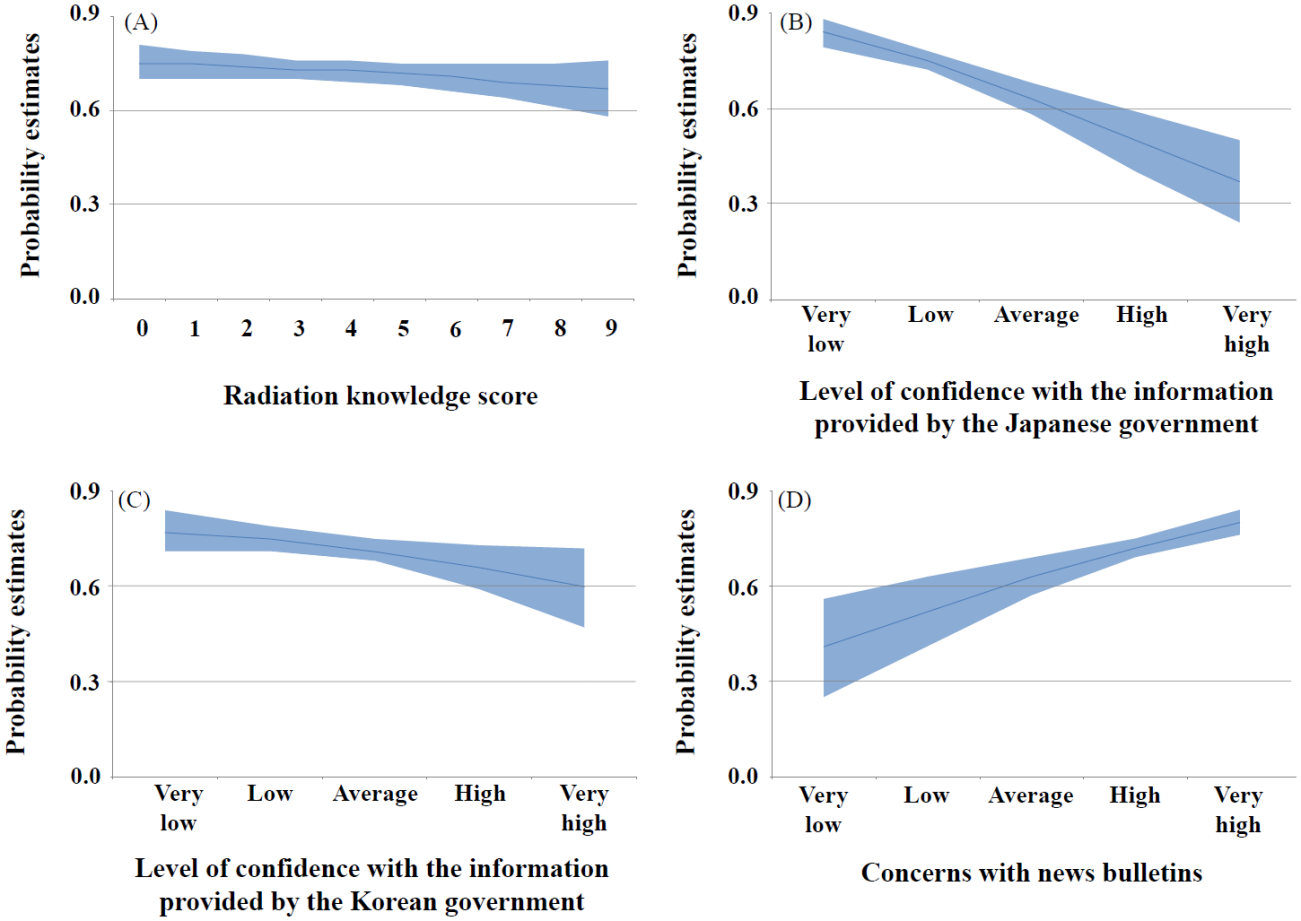
Changes in the purchase intentions of Japanese seafood with non-detectable levels of radiation per the radiation knowledge score, the level of confidence in the information provided by the Japanese and Korean governments, and concerns with news bulletins. The x-axis shows the radiation knowledge score (A), level of confidence in the information provided by the Japanese government (B), level of confidence in the information provided by the Korean government (C), and the concerns with news bulletins (D). The y-axis denotes probability estimates of non-intention to purchase Japanese seafood with non-detectable levels of radiation after adjusting for significant variables in the multivariate model. The 95% confidence interval on the estimates is shown in blue shading. The increase in radiation knowledge score, as well as the levels of confidence in the information by Korean and Japanese governments decreased the probability of non-purchasing intentions of Japanese seafood with non-detectable levels of radiation (A, B, C). The increased concerns with news bulletins were associated with an increased probability of non-purchasing intentions (D).

When asked whether they would purchase Japanese seafood when the level of radiation in the food was non-detectable, 68.8% of the respondents answered that they “would not purchase.” To identify variables relating to such response, the reference category of the response was set as “will purchase” and a multinomial logistic regression was conducted for the remaining two responses (i.e., I do not know, would not purchase). The results of the univariate analysis showed that the variables related to the negative response (i.e., would not purchase) to the purchase intentions were association with radioactivity (e.g., the Fukushima nuclear power plant accident), concerns with the health effects from radiation exposure, a low radiation knowledge score, low confidence level with the government and mass media when providing radioactive information, low confidence in the management of radioactive contamination of food, and concerns with news bulletins. While there was an increased tendency for purchase intentions in respondents with higher education levels (p = 0.123), difference in purchase intentions per sex and age was not found (Table 6). The multivariate analysis revealed that concerns with the health effects of radiation exposure, a low radiation knowledge score, a low confidence level with the government when providing radioactive information, and concerns with news bulletins were identified as significant variables (Table 6). Predicted probabilities of the decrease in purchase intentions of Japanese seafood showed limited changes per the radiation knowledge score, but large changes per the concerns with news bulletins and confidence in the Japanese government (Fig 2).

**Table 6:** Factors associated with non-intention to purchase Japanese seafood with non-detectable levels of radiation.

Variables^a^	Univariate		Multivariate	
	Relative Risk (95% CI)	*p*-value	Relative Risk (95% CI)	*p*-value
Received information				
Yes	0.84 (0.56, 1.27)	0.420	-	
Association with radioactivity		0.021		
Nuclear weapons and nuclear power plants	1.57 (0.65, 3.81)	0.320	-	
Fukushima nuclear power plant accident	3.08 (1.23, 7.68)	0.016	-	
Radon	1.52 (0.15, 15.29)	0.722	-	
Other	192311.8 (0, ∞)	0.976	-	
Health implications when exposed		0.808		
Child deformity	1.13 (0.71, 1.79)	0.616	-	
Genetic diseases	1.08 (0.55, 2.11)	0.830	-	
Early death due to mysterious disease	0.77 (0.38, 1.57)	0.475	-	
Other	2.04 (0.26, 16.07)	0.497	-	
Concerned with health implications from radiation exposure		<0.001		0.005
Yes	2.99 (1.82, 4.91)	< 0.001	2.40 (1.39, 4.12)	0.002
Do not care	1.43 (0.87, 2.36)	0.157	1.22 (0.71, 2.09)	0.474
Source of radioactive material included in agricultural products		0.451		
There cannot be any radioactive material	1.21 (0.35, 4.21)	0.766	-	
Radioactive material from nuclear power plants (Fukushima and Korea)	1.35 (0.85, 2.16)	0.208	-	
Radioactive material residues from nuclear weapon tests in the past	0.99 (0.28, 3.49)	0.986	-	
Do not know	0.87 (0.50, 1.53)	0.631	-	
Important factors when purchasing food		0.053		
Freshness and expiry date	1.28 (0.73, 2.24)	0.392	-	
Origin	3.31 (1.44, 7.60)	0.005	-	
Safety	2.03 (1.02, 4.04)	0.043	-	
Price	1.06 (0.28, 4.04)	0.931	-	
Do not know as no purchase made	1.14 (0.23, 5.59)	0.875	-	
Foods that should have origins checked		0.300		
Baby food	0.64 (0.27, 1.52)	0.314	-	
Seafood	0.84 (0.53, 1.34)	0.462	-	
Rice	0.90 (0.34, 2.40)	0.833	-	
Specific products	0.41 (0.18, 0.96)	0.040	-	
Awareness of updated information on the MFDS’s website				
Yes	0.82 (0.50, 1.35)	0.444	-	
Utilization of information from MFDS				
Yes	0.78 (0.48, 1.27)	0.318	-	
Radiation knowledge score	0.86 (0.78, 0.94)	0.002	0.86 (0.78, 0.96)	0.007
Confidence in the information provided by the Japanese government	0.38 (0.30, 0.49)	< 0.001	0.57 (0.43, 0.76)	< 0.001
Confidence in the information provided by the Korean government	0.39 (0.30, 0.49)	< 0.001	0.58 (0.43, 0.79)	< 0.001
Concerns with news bulletins	2.04 (1.55, 2.68)	< 0.001	1.74 (1.29, 2.34)	< 0.001
Confidence in the information provided by the mass media	0.67 (0.52, 0.87)	0.002	-	
Confidence in the management of radioactive contamination of food	0.43 (0.33, 0.57)	< 0.001	-	
Sex				
Female	1.12 (0.75, 1.68)	0.575	-	
Age		0.402		
30s	1.16 (0.60, 2.24)	0.653	-	
40s	1.44 (0.73, 2.81)	0.291	-	
50s	0.81 (0.43, 1.51)	0.499	-	
60s and older	1.14 (0.53, 2.44)	0.745	-	
Education level				
Post-secondary graduate	0.68 (0.41, 1.11)	0.123	-	
Occupation		0.672		
Professional	0.83 (0.45, 1.51)	0.533	-	
Student	0.69 (0.30, 1.58)	0.386	-	
Self-employed and other	0.69 (0.37, 1.30)	0.253	-	
Age of family members		0.141		
Elementary school and younger	2.07 (1.07, 4.00)	0.031	-	
Middle or high school students	1.64 (0.88, 3.08)	0.120	-	
Adults (over 20 years)	1.28 (0.75, 2.19)	0.364	-	
Number of observations			1040	
Hosmer-Lemeshow goodness of fit			0.110	

^a^Reference categories for each independent variable: experience of information receipt (no), association with radioactivity (hospitals), health influence when exposed to radiation (cancer), health influence concerns from radiation (no), type of radiation included in agricultural products (natural radiation), important factors in food purchase (quality and taste), products whose origin is checked (all products), awareness of updated information on the MFDS’s website (No), utilization of information from MFDS (no), sex (male), age (20s), education levels (high school or below), occupation (housewives), age of family members (none).

#### Agreement with maintaining the current level of import restraints of Japanese seafood

**Fig 3:**
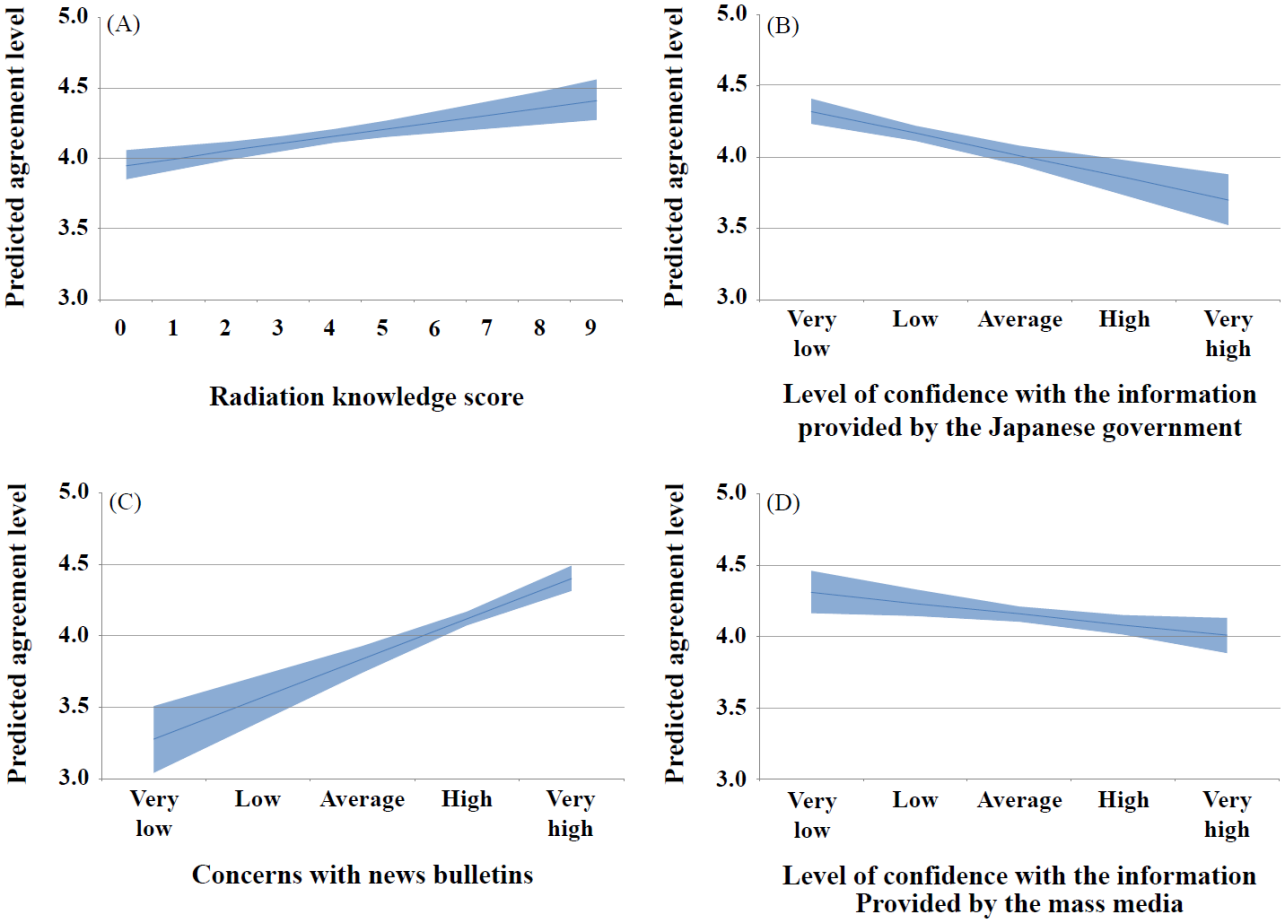
Changes in the agreement level with the maintenance of current import restraints of Japanese seafood per radiation knowledge score, the level of confidence in the information provided by the Japanese and the level of confidence in the information provided by the mass media. The x-axis denotes radiation knowledge score (A), level of confidence in the information provided by the Japanese government (B), concerns with the news bulletins (C), and the level of confidence in the information provided by the mass media (D). The y-axis shows estimates on the level of agreement of maintaining the current import restraints of Japanese seafood after adjusting for significant variables in the multivariate model. The 95% confidence interval on the estimates is shown in blue shading. Higher radiation knowledge scores and concerns with news bulletins were associated with increased agreement with maintaining the current import restraints of Japanese seafood (A, C), and higher levels of confidence in the Japanese government and the mass media were associated with the decreased agreement with maintaining the current import restraint levels on Japanese seafood (B, D).

The levels of agreement with maintaining the current levels of import restraints on Japanese seafood were measured using a 5-point Likert scale (i.e., *not at all (relaxation of import restraints)* = 1, *no* = 2, *average* = 3, *yes* = 4, and *definitely yes* = 5). Participants scored an average of 4.1. A linear regression analysis was conducted to identify variables related to continuing import restraints. The results of the univariate analysis showed that various variables were related to agreement with the current import restraints including the association with radioactivity, concerns with the health effects of radiation exposure, the source of radioactive material included in agricultural products, important factors considered when purchasing food (e.g., origin, safety), types of food that have their origins checked (e.g., baby food), a high radiation knowledge score, low confidence level with the government and mass media when providing radioactive information, low confidence in the management of radioactivity in food, concerns with news bulletins, age (e.g., 30–50s), occupation (e.g., housewives) and living with a child (Table 7). The multivariate analysis revealed that types of food that have their origins checked, radiation knowledge score, low confidence in the Japanese government and mass media on radiation information, concerns from news bulletins, and occupation were still significant (Table 7). Strong agreement with the current import restraints were observed for high radiation knowledge and low confidence in information provided by the mass media; however, their changes of the level of agreement were relatively limited compared to changes associated with concerns with news bulletins and confidence in the Japanese government (Fig 3).

**Table 7:** Factors associated with agreement with the current import restraints of Japanese seafood.

Variables^a^	Univariate		Multivariate	
	Coefficient (95% CI)	*p*-value	Coefficient (95% CI)	*p*-value
Received information				
Yes	0.04 (-0.07, 0.16)	0.458	-	
Association with radioactivity		0.020		
Nuclear weapons and nuclear power plants	0.09 (-0.25, 0.44)	0.598	-	
Fukushima nuclear power plant accident	0.26 (-0.08, 0.61)	0.133	-	
Radon	0.54 (-0.16, 1.24)	0.132	-	
Other	0.44 (-0.41, 1.28)	0.311	-	
Health implications when exposed		0.932		
Child deformity	-0.03 (-0.16, 0.09)	0.626	-	
Genetic diseases	0.01 (-0.17, 0.20)	0.875	-	
Early death due to mysterious disease	-0.03 (-0.25, 0.18)	0.752	-	
Other	0.12 (-0.30, 0.55)	0.562	-	
Concerned with health implications from radiation exposure		< 0.001		
Yes	0.26 (0.12, 0.39)	< 0.001	-	
Do not care	0.05 (-0.10, 0.20)	0.499	-	
Source of radioactive material included in agricultural products		0.045		
There cannot be any radioactive material	-0.31 (-0.64, 0.01)	0.061	-	
Radioactive material from nuclear power plants (Fukushima and Korea)	-0.03 (-0.15, 0.09)	0.625	-	
Radioactive material residues from nuclear weapon tests in the past	0.30 (-0.08, 0.67)	0.118	-	
Do not know	-0.16 (-0.32, 0.01)	0.061	-	
Important factors when purchasing food		< 0.001		
Freshness and expiry date	-0.04 (-0.21, 0.12)	0.592	-	
Origin	0.25 (0.05, 0.44)	0.012	-	
Safety	0.27 (0.09, 0.46)	0.004	-	
Price	-0.17 (-0.52, 0.18)	0.337	-	
Do not know as no purchase made	0.06 (-0.40, 0.51)	0.804	-	
Foods that should have origins checked		0.002		< 0.001
Baby food	-0.49 (-0.75, -0.23)	< 0.001	-0.47 (-0.72, -0.23)	< 0.001
Seafood	0.06 (-0.06, 0.19)	0.315	0.10 (-0.02, 0.22)	0.114
Rice	-0.11 (-0.36, 0.14)	0.381	0.06 (-0.18, 0.30)	0.632
Specific products	0.04 (-0.24, 0.32)	0.776	0.21 (-0.05, 0.48)	0.117
Awareness of updated information on the MFDS’s website				
Yes	0.10 (-0.04, 0.25)	0.160	-	
Utilization of information from MFDS				
Yes	-0.03 (-0.17, 0.12)	0.703	-	
Radiation knowledge score	0.04 (0.01, 0.07)	0.003	0.05 (0.03, 0.08)	< 0.001
Confidence in the information provided by the Japanese government	-0.19 (-0.25, -0.13)	< 0.001	-0.15 (-0.21, -0.09)	< 0.001
Confidence in the information provided by the Korean government	-0.18 (-0.24, -0.12)	< 0.001	-	
Concerns with news bulletins	0.32 (0.25, 0.40)	< 0.001	0.28 (0.20, 0.35)	< 0.001
Confidence in the information provided by the mass media	-0.14 (-0.20, -0.07)	< 0.001	-0.08 (-0.14, -0.01)	0.019
Confidence in the management of radioactive contamination of food	-0.18 (-0.25, -0.11)	< 0.001	-	
Sex				
Female	0.00 (-0.11, 0.11)	0.954	-	
Age		0.021		
30s	0.21 (0.03, 0.38)	0.021	-	
40s	0.25 (0.08, 0.43)	0.004	-	
50s	0.25 (0.08, 0.43)	0.004	-	
60s and older	0.10 (-0.11, 0.31)	0.343	-	
Education level				
Post-secondary graduate	0.01 (-0.11, 0.14)	0.846	-	
Occupation		0.032		0.016
Professional	-0.12 (-0.27, 0.04)	0.133	-0.17 (-0.32, -0.03)	0.020
Student	-0.33 (-0.54, -0.11)	0.003	-0.33 (-0.53, -0.12)	0.002
Self-employed and other	-0.15 (-0.31, 0.02)	0.086	-0.18 (-0.34, -0.02)	0.028
Age of family members		0.013		
Elementary school and younger	0.19 (0.01, 0.37)	0.040	-	
Middle or high school students	0.11 (-0.06, 0.29)	0.195	-	
Adults (over 20 years)	-0.04 (-0.20, 0.12)	0.614	-	
Number of observations			996	
Hosmer-Lemeshow goodness of fit			0.133	

^a^Reference categories for each independent variable: experience of information receipt (no), association with radioactivity (hospitals), health influence when exposed to radiation exposure (cancer), health influence concerns from radiation (no), type of radiation included in agricultural products (natural radiation), important factors in food purchase (quality and taste), products whose origin is checked (all products), awareness of updated information on the MFDS’s website (no), utilization of information from MFDS (no), sex (male), age (20s), education levels (high school or below), occupation (housewives), age of family members (none).

## Discussion

To understand the factors influencing the risk perception on radiation-related seafood and opinions on the regulatory policy, this study examined changes in seafood purchase frequency after the Fukushima accident, purchase intentions of Japanese seafood with non-detectable levels of radiation, and agreement with the current levels of import restraints of Japanese seafood as key questions of interest.

In this study, most respondents decreased their purchases of seafood after the Fukushima accident and would not purchase seafood, even if the levels of radiation in the food were non-detectable. These results are similar to a recent study [18] reporting that 77% of respondents did not purchase Japanese food products after the Fukushima accident, and showed that the majority of consumers, despite more than 3 years having passed since the Fukushima accident, are still avoiding Japanese seafood. In a similar study that surveyed Americans, 33% responded that they saw decreases in purchasing Asian seafood products after the Fukushima accident and 63% of the respondents thought that there would be threats to consumer health from Asian seafood [3]. Such differences between the attitudes towards seafood purchase by U.S. and Korea seem to result from differences in survey questions (e.g., Japanese food vs. Asian food), differences in the level of concerns owing to the difference in physical distance from Japan, and differences in food consumption habits such that annual fish consumption in Korea (58.4 kg per capita) is higher than that in the U.S. (23.7 kg per capita) [19]; however, the general negative perception of seafood from neighboring oceans after the Fukushima accident seems to be similar.

Relating to information searching activities and levels of radiation knowledge, a previous study [18] reported that 19.1% of the respondents searched for information relating to the Fukushima accident; moreover, this study reported similar results with 17.4% of the respondents knowing that the MFDS website was providing information on the management of radioactivity in imported food on a daily basis, and 17.7% of the respondents referring to the information provided by the MFDS when purchasing food products. The radiation knowledge scores averaged 3.63 out of 9, showing similar or lower levels to the score of 3.1 out of 6 in the previous study. While there were differences in the survey questions between the studies, this shows that despite a high level of interest and concern on the exposure to radiation and the effects from food products following the Fukushima accident, the opportunities to encounter and to collect correct information remain limited.

In most studies on risk perception including food safety, women were found to be more sensitive to risk than men were [14,20–22]. In an Australian study, the odds ratio of women’s level of concern about food safety and quality were 1.69 (p < 0.05), indicating women’s concern was significantly higher than men’s concern [22]; other perception studies showed that women were more concerned than men about bacteria in food (women: 80.2%, men: 70.8%), food additives (women: 81.0%, men: 73.2%) and pesticide residues in food (women: 80.1%, men: 70.6%) [14]. This study also found that women were more concerned about the health risks of daily exposure to radiation; however, the difference between men and women was 6%, and was not statistically significant for the key questions about risk perception in this study including seafood purchases, purchase intentions, and agreement with maintaining the current import restraints of Japanese seafood. While a previous study on purchasing Japanese food products [18] showed statistically significant differences by sex, the size of the difference was also around 5%, not representing a large difference in the risk perception compared to other harmful substances; this might indicate that a radiation accident in a neighboring nation has had a large influence on the citizens regardless of sex.

**Fig 4:**
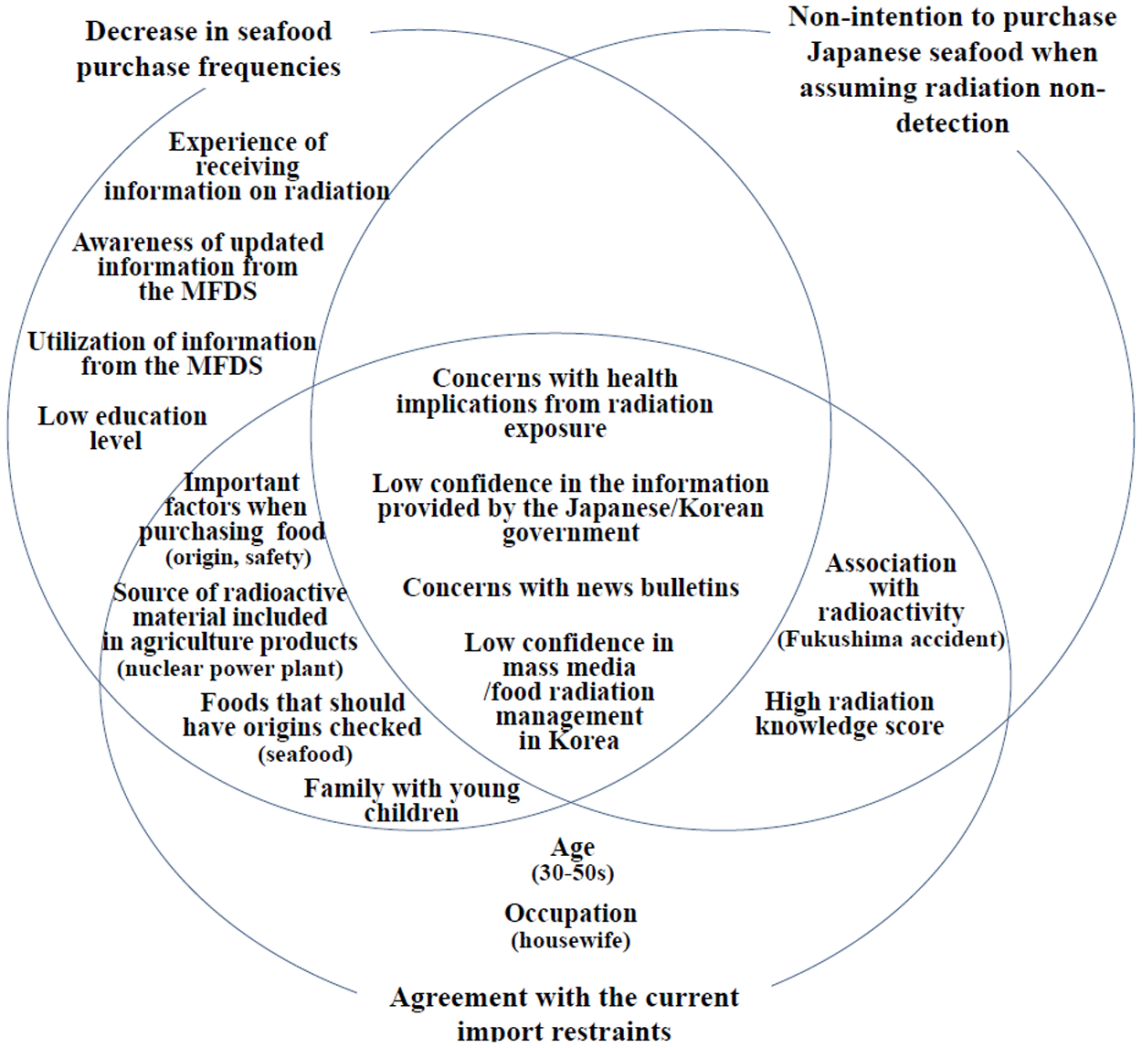
Factors associated with the purchase tendencies, intentions to purchase Japanese seafood, and agreement with the current import restraints after the Fukushima nuclear power plant accident.

Common variables associated with conservative responses to Japanese seafood from the univariate analyses in this study included concerns about health effects from radiation exposure, low confidence level in the government and mass media when providing radioactive information, and concerns with news bulletins reporting on the increase in discovery of small amounts of radioactive material (Fig 4).

Therefore, it is important to deliver information regarding not only radiation exposure, but also the potential health effects from that exposure, to better communicate the risks of radiation-related food safety issues. This relies on trusting the government and the mass media. Awareness and utilization of information provided by the MFDS was related to the decrease in seafood purchase frequency after the Fukushima accident; however, it was not related to the purchase intentions of Japanese seafood with non-detectable levels of radiation or agreement with maintaining the current import restraints of Japanese seafood. Therefore, perhaps respondents with more concern for radioactive contamination of seafood were more interested in information gathering, rather than adhering to the information provided by the MFDS. While there was an increasing tendency in the purchase frequency for respondents with higher education levels, the agreement with maintaining the current levels of import restraints of Japanese seafood was not related to education levels. Similarly, a higher radiation knowledge score was associated with the increase of purchase intentions for Japanese seafood with non-detectable levels of radiation, but strong agreement with maintaining the current import restraints. This implies a low level of confidence on the management of Japanese seafood and conservative tendencies towards food safety, regardless of education levels. Age of family members was not statistically significantly associated with any key questions in the multivariate analyses (p-values = 0.064–0.194), which might be masked by other factors. However, this variable could be utilized to identify people who are more sensitive to perceived risk in a simpler and objective manner.

Our results cannot be generalized to risk perception of all radiation-related food because the major survey questions focused on Japanese seafood following the Fukushima accident. However, since seafood consumption is very closely tied to daily life in Korea, where we revealed differences of risk perceptions per a complex mixture of individual characteristics and the surrounding environment, our findings could reflect typical characteristics of the public’s responses to food safety regarding radiation exposure. In addition, this survey was the first study to examine public opinion on import restraints of the potential radioactive contamination of food in South Korea. As South Korea is a neighboring country of Japan, these national survey results could help policy-makers understand the public's risk perception of potential radioactive contamination of food and predict their responses to food consumption and import restraint policies due to nuclear accidents in neighboring countries. While this study enables the understanding of risk perception on the potential radioactive contamination of food at a single time point after the Fukushima accident, it is limited in assessing the trends of changes in risk perceptions thereafter. As such, further studies are required that address long-term risk perception changes and on identifying influencing factors thereof. We are planning to conduct the same survey including additional questions exploring responses to different types of information sources, and expect to present a long-term trend of risk perception and purchase behavior of Fukushima-related food, which could provide insight on implementing communication strategies for radiation-related food safety.

## Conclusions

Our results have real-world implications for the public’s responses to food safety including consumption behavior and import restraints due to nuclear accidents in neighboring countries. This study revealed a lingering high level of concern regarding risk of Japanese seafood in South Korea following the Fukushima accident. The risk perception was associated with a complex mixture of individual characteristics such as education level, radiation knowledge, and confidence in the government, and the surrounding environment such as news bulletins and one’s family members. Therefore, effective risk communication requires the understanding of baseline levels of awareness and the heterogeneity of risk perceptions per communication targets. In particular, confidence in information providers including government agencies has a large influence on risk perception of radioactive contamination of food as well as consumption behavior and policy directions; thus, trust needs to be built between the provider and the recipient of information. Moreover, bilateral information communication and the development of education programs wherein the public, especially families with young children, can participate should be encouraged for dissemination of correct information on radiation-related food safety.

## Supporting information

S1 Appendix Survey questionnaire in Korean. (DOCX)

S2 Appendix Survey questionnaire in English. (DOCX)

## References

[1] Hosono H, Kumagai Y, Sekizaki T. Development of an information package of radiation risk in beef after the Fukushima Daiichi nuclear power plant accident In: Nakanishi T, Tanoi K, editors. Agricultural implications of the Fukushima nuclear accident. Japan: Springer; 2013 pp. 187–204. doi: 10.1007/978-4-431-54328-2_17

[2] Kimura AH, Bain C, Ransom E, Higgins V. Standards as hybrid forum: Comparison of the post-Fukushima radiation standards by a consumer cooperative, the private sector, and the Japanese government. IJSAF. 2013 1 1;20(1): 11–29.

[3] McKendree MG, Ortega DL, Widmar NO, Wang HH. Consumer perceptions of seafood industries in the wake of the deepwater horizon oil spill and Fukushima Daiichi nuclear disaster. Staff report. Michigan: Michigan State University, Department of Agricultural, Food, and Resource Economics; 2013. pp. 1–22.

[4] Ministry of Food and Drug Safety. Government bans import of all marine products from eight prefectiures near Fukushima. 2013 Sep 6. Korean. http://www.mfds.go.kr/index.do?mid=977&pageNo=14&seq=21213&cmd=v.

[5] Badrie N, Gobin A, Dookeran S, Duncan R. Consumer awareness and perception to food safety hazards in Trinidad, West Indies. Food Control. 2006;17(5): 370–377. doi: 10.1016/j.foodcont.2005.01.003

[6] Miles T. Japan takes South Korea to WTO over Fukushima-related food import restrictions. Reuters. 2015 May 21. http://www.reuters.com/article/us-japan-southkorea-wto-nuclear-idUSKBN0O615F20150521.

[7] Jung H. Gyeongnam consumer organizations “Be banned Japanese seafood imports”. Yonhapnews. 2013 Oct 1. Korean. http://news.naver.com/main/read.nhn?mode=LSD&mid=sec&sid1=102&oid=001&aid=0006511319.

[8] The voice of Seoul. All areas of Japan's seafood radioactive contaminated, 'Let's ban on imports'. The voice of Seoul. 2013 Sep 8. Koran. http://www.amn.kr/sub_read.html?uid=10781.

[9] Kim J. Japanese fish worry consumers. The Korea Times. 2013 Aug 29. http://koreatimes.co.kr/www/news/nation/2013/08/113_141912.html.

[10] Wakatsuki Y. New radioactive water leak at Japan's Fukushima Daiichi plant. CNN. 2014 Feb 20. http://edition.cnn.com/2014/02/19/world/asia/japan-fukushima-daiichi-water-leak/.

[11] Brewer MS, Rojas M. Consumer attitudes toward issues in food safety. J of Food Saf. 2008;28(1): 1–22. doi: 10.1111/j.1745-4565.2007.00091.x

[12] Costa-Font M, Gil JM, Traill WB. Consumer acceptance, valuation of and attitudes towards genetically modified food: Review and implications for food policy. Food Policy. 2008;33(2): 99–111. doi: 10.1016/j.foodpol.2007.07.002

[13] Ergönül B. Consumer awareness and perception to food safety: A consumer analysis. Food Control. 2013;32(2): 461–471. doi: 10.1016/j.foodcont.2013.01.018

[14] Knight A, Warland R. The relationship between sociodemographics and concern about food safety issues. J Consum Aff. 2004;38(1): 107–120. doi: 10.1111/j.1745-6606.2004.tb00467.x

[15] Hosono H, Iwabuchi M, Kumagai Y, Sekizaki T. Japanese consumers’ altruistic attitude and food choice: two years after Fukushima accident In: Hongladarom S, editor. Food security and food safety for the twenty-first century. Springer Singapore; 2015 pp. 319–333. doi: 10.1007/978-981-287-417-7_28

[16] Peterson H, Yamaura K. Ambiguity aversion and preferences for food origin post Fukushima nuclear disaster. In: 2014 annual meeting, July 27–29, 2014, Minneapolis, Minnesota: Agricultural and Applied Economics Association; 2014.

[17] Sawada M, Aizaki H, Sato K. Japanese consumers’ valuation of domestic beef after the Fukushima Daiichi Nuclear Power Plant accident. 2014;80: 225–235. doi: 10.1016/j.appet.2014.05.018 24859113 10.1016/j.appet.2014.05.018

[18] Kim NH, Cho TJ, Kim YB, Park BI, Kim HS, Rhee MS. Implications for effective food risk communication following the Fukushima nuclear accident based on a consumer survey. Food Control. 2015;50: 304–312. doi: 10.1016/j.foodcont.2014.09.008

[19] Food and Agriculture Organization of the United Nations (FAO). The state of world fisheries and aquaculture: Contributing to food security and nutrition for all. Rome: FAO; 2016.

[20] Kim K, Kim HJ, Song DJ, Cho YM, Choi JW. Risk perception and public concerns of electromagnetic waves from cellular phones in Korea. Bioelectromagnetics. 2014;35(4): 235–244. doi: 10.1002/bem.21836 24500860 10.1002/bem.21836

[21] Kung YW, Chen SH. Perception of earthquake risk in Taiwan: effects of gender and past earthquake experience. Risk Anal. 2012;32(9): 1535–1546. doi: 10.1111/j.1539-6924.2011.01760.x 22300232 10.1111/j.1539-6924.2011.01760.x

[22] Taylor AW, Coveney J, Ward PR, Dal Grande E, Mamerow L, Henderson J, et al The Australian Food and Trust Survey: Demographic indicators associated with food safety and quality concerns. Food Control. 2012;25(2): 476–483. doi: 10.1016/j.foodcont.2011.11.003

