# Supplementary material for: Factors associated with the risk perception and purchase decisions of Fukushima-related food in South Korea
Source: PLoS One. 2017 Nov 8;12(11):e0187655. doi: 10.1371/journal.pone.0187655 (PMC5695610; doi:10.1371/journal.pone.0187655)
Supplement: S1 Appendix — (DOCX) [file pone.0187655.s001.docx]

| 후쿠시마 원전사고 이후 방사능 오염식품에 대한 국민의 불안감이 컸습니다. 국내 원전에서도 여러 가지 문제가 드러나 국민의 불안감이 가중되고 있습니다. 본 조사는 국민이 방사선과 그 위험에 대해 어떻게 생각하고 판단하는지를 파악하여 정부의 방사선 안전정책에서 소통을 중시하도록 정책자료로 제공하려는 것입니다.  2014. 11.  한국소비자연맹 |
| --- |

**S1 Appendix. Survey questionnaire in Korean**

**Ⅰ. 방사능물질에 대한 인식 조사**

1. 방사선이나 방사능물질 등에 대해 교육 등 정보제공을 받은 적이 있습니까?

| ① 그렇다(1-1로 가세요) | ② 아니다(2로 가세요) |
| --- | --- |

1-1. 정보제공을 받은 경험이 있다면 어디서 정보를 **가장 많이** 받았습니까?

| ① 매스컴(TV, 신문, 라디오 등) | ② 강연, 세미나 등 | ③ 학교 교육 |
| --- | --- | --- |
| ④ 인터넷, SNS 등 | ⑤ NGO 등의 교육 프로그램 | ⑥ 정부 홈페이지 |
| ⑦ 친구 등 아는 사람을 통해 | ⑧ 정부나 공공기관 유인물 | ⑨ 기타 ( ) |
| ⑩ 방사선에 관심 없음 |  |  |

2. 다음 식품의 유해인자 중 **매우 위험**하다고 느끼는 것 **3가지만 선택(○)**해 주세요

| 구분 | 위험 | 구분 | 위험 |
| --- | --- | --- | --- |
| 잔류농약 |  | GMO(유전자변형식품) |  |
| 노로바이러스 |  | 메틸수은 |  |
| 광우병 |  | 식중독세균(대장균, O-157등) |  |
| 방사능 오염 식품 |  | 중금속(납) |  |
| 식품첨가물 |  | 방사선조사식품 |  |

3. ‘방사능’이란 말을 들을 때 **가장 먼저** 떠오르는 생각은 무엇입니까?

| ① 병원 | ② 핵무기 | ③ 원자력발전소 |
| --- | --- | --- |
| ④ 후쿠시마 원전사고 | ⑤ 라돈 | ⑥ 기타 ( ) |

4. 방사선에 노출(피폭)된 경우 가장 우려하는 건강 영향은 무엇입니까?

| ① 암(백혈병 포함) 발생 | ② 기형아 | ③ 유전병 |
| --- | --- | --- |
| ④ 괴질로 인한 조기 사망 | ⑤ 모르겠다 | ⑥기타( ) |

5. 귀하는 일상생활에서 방사선(방사능물질)에 의해 건강에 위협을 받고 있다고 생각하십니까?

| ① 그렇다(5-1로 가세요) | ② 아니다(6으로 가세요) | ③ 신경 쓰지 않는다(6으로 가세요) |
| --- | --- | --- |

5-1. 위 문항에서 ①을 응답하신 이유는 무엇입니까?

| ① 다른 사람들도 그렇게 생각하고 있기 때문에 |
| --- |
| ② 방사선 위험에 대해 과학자도 잘 모르기 때문에 |
| ③ 알게 모르게 주변에서 방사선이 사용되고 있기 때문에 |
| ④ 후쿠시마 원전이나 우리 원전에서 방사능물질이 계속 나오고 있기 때문에 |
| ⑤ 방사선은 아무리 작아도 무조건 위험하기 때문에 |
| ⑥ 기타 ( ) |

| 자연계에는 라돈, 칼륨-40과 같은 천연방사능 물질이 있습니다. 라돈은 땅 속의 천연방사능 물질이 공기 중으로 스며 나온 것으로서 어디나 존재합니다. 모든 식품에는 칼륨-40이 들어 있습니다. 국제선 비행기 탑승 중에는 우주방사선을 피폭합니다. 우리국민의 자연방사선 피폭 수준은 연간 약 3.0 밀리 시버트(mSv)입니다. 후쿠시마 원전 사고 때문에 당시 등으로 방출된 요오드-131, 세슘은 인공 방사능입니다. |
| --- |

| * 시버트(Sv): 사람이 방사선에 노출된 양을 재는 단위  * 베크렐(Bq): 방사능의 양을 재는 단위 |
| --- |

6. 우리 농산물을 사용한 밥이나 김치에도 방사능이 들어 있을 수 있다고 합니다. 어떤 방사능이 **가장** 많다고 생각하십니까?

| ① 방사능이 있을 리가 없다 | ② 후쿠시마 원전에서 날아온 방사능 |
| --- | --- |
| ③ 과거 지상핵실험 낙진 방사능 | ④ 천연방사능 |
| ⑤ 우리나라 원전에서 나온 방사능 | ⑥ 모르겠다 |

**II. 식품 구입에 대한 조사**

7. 식품 구입시 무엇을 중요하게 생각합니까?

| ① 품질(맛) | ② 신선도 | ③ 유통기한(품질유지기한) | | | ④ 원산지 |
| --- | --- | --- | --- | --- | --- |
| ⑤ 안전성(알러지, 방사능물질, 식품첨가물 등) | | | ⑥ 가격 | ⑦ 구입하지 않아 모른다 | |

8. 원산지를 확인하고 구입하는 식품은 무엇입니까?

| ① 모든 식품 | ② 유아식 | ③ 수산물 | ④ 쌀 | ⑤ 특정 식품( ) |
| --- | --- | --- | --- | --- |

9. 수산물 구입 경향은 어떻습니까?

| ① 국내산만 구입한다 |
| --- |
| ② 국내산, 수입산 상관없이 구입한다. |
| ③ 일본산만 기피한다 |
| ④ 국내산이든 수입산이든 수산물은 아예 구입하지 않는다 |
| ⑤ 국내산과 수입산의 구분이 어려워 아예 구입하지 않는다 |

10. 후쿠시마 원전 사고 이후 현재 수산물의 구입 빈도가 어떻습니까?

| ① 사고 전과 차이가 없다(11로 가세요) | ② 수산물 구입 빈도가 줄었다(10-1로 가세요) |
| --- | --- |
| ③ 수산물 구입 빈도가 늘었다(11로 가세요) | ④ 수산물 구입을 아예 않는다(10-1로 가세요) |

10-1. 문항 10번에서 ②, ④를 응답하신 이유는 무엇입니까? **2개** 고르세요.

| ① 여전히 수입산 수산물 중 일본산은 있기 때문이다 |
| --- |
| ② 방사능물질이 미량이라도 수산물에서 검출되기 때문이다 |
| ③ 일본 정보를 신뢰할 수 없기 때문이다 |
| ④ 원산지 표시를 신뢰할 수 없기 때문이다 |
| ⑤ 한국 식약처의 검사 결과를 신뢰할 수 없기 때문이다 |
| ⑥ 한국 식약처의 검사가 미진하기 때문이다 |

11. 현재 일본산 수입 수산물의 방사능 수준은 거의 불검출 수준입니다.

이럴 경우 앞으로 일본산 생선을 구입하시겠습니까?

| ① 구입한다 (11-2로 가세요) | ② 구입하지 않는다 (11-1로 가세요) |
| --- | --- |
| ③ 모르겠다 (11-1로 가세요) |  |

11-1. ‘구입하지 않는다 또는 모르겠다’라고 답한 이유는 무엇입니까?

| ① 안전기준 자체가 잘못된 것이어서 |
| --- |
| ② 정부가 무언가 숨기는 것이 있을 것 같아서 |
| ③ 모두 검사하는 것이 아니므로 내가 산 식품의 방사능이 높을 수 있기 때문에 |
| ④ 2011년 해양으로 방출된 방사능이 태평양을 돌아올 때가 되었기 때문에 |

11-2. ‘구입한다’로 답한 이유는 무엇입니까?

| ① 불검출 수준은 안전하다고 생각하기 때문에 |
| --- |
| ② 정부의 관리를 신뢰하기 때문에 |
| ③ 일본에서도 문제의 해역관리를 잘하고 있는 것 같기 때문에 |
| ④ 수산물 방사능에 대한 걱정이 처음부터 불필요한 것이기 때문에 |

12. 후쿠시마 원전사고 이후 정부에서 수입식품 방사능관리 현황을 식약처 홈페이지에서 제공하고 있는 사실을 알고 계십니까?

| ① 그렇다 | ② 아니다 |
| --- | --- |

12-1. 식품 구입시 식약처에서 제공하는 위해정보를 참고하십니까?

| ① 그렇다 | ② 아니다 |
| --- | --- |

**III. 방사선과 방사능에 대한 지식 수준 조사**

13. 다음 사항에 대해 어떻게 알고 계십니까?(√표시 해주세요)

| **구분** | **①그렇다** | **②아니다** | **③ 잘모른다** |
| --- | --- | --- | --- |
| 가. 자연계에는 천연방사능이 있어 모든 물질은 미량 의 방사능을 가지고 있다 |  |  |  |
| 나. 자연방사선량에 노출되는 양은 지역, 주택유형, 습관 등에 따라 다를 수 있다 |  |  |  |
| 다. 자연방사선이나 인공방사선(원전사고 등으로 방출되는 방사선, X선)이나 다 같은 방사선이다 |  |  |  |
| 라. 천연방사능은 인공방사능보다 덜 해롭다 |  |  |  |
| 마. 요오드-131, 세슘-137은 인공 방사능 물질이다 |  |  |  |
| 바. 정부는 인체에 안전한 방사능 허용기준을 두어 관리 한다 |  |  |  |
| 사. 단기간에 방사선을 대량 피폭하지 않는 이상, 방사선으로 인해 건강을 해칠 가능성은 매우 낮다 |  |  |  |
| 아. 몸에 들어 온 방사능은 자연붕괴하거나 신진대사(소변, 대변 등)로 배설되어 결국 없어진다 |  |  |  |
| 자. 방사성 세슘 100Bq(현행기준)이 검출된 생선을 일상적으로 섭취해도 평소 받는 방사선량을 별로 증가하지 않는다 |  |  |  |

| 우리나라의 식품 방사능 기준으로 **세슘은 모든 식품에 1 kg 당 100** **베크렐(100 Bq/kg)**로 정해 관리하고 있습니다 |
| --- |

14. 생선 기준치 **1 kg 당 100 베크렐**보다 휠씬 낮은 **10베크렐이 검출**되었다면 어떻게 하시겠습니까?

| ① 10베크렐이 아니라 1베크렐도 방사능은 싫어 구입하지 않는다 |
| --- |
| ② 기준치 이하더라도 인체에 위해하다고 생각하기 때문에 구입하지 않는다 |
| ③ 기준치 이하면 안전하므로 구입한다 |
| ④ 방사능이 얼마나 있는지도 모르는 다른 식품에 비해 오히려 안심되어 구입한다 |

**IV. 방사능 물질 관리 신뢰도 및 안전관리 정책 인지**

15. 귀하는 일본 정부 방사능 정보에 대한 신뢰는 어느 정도 입니까?

① 매우 신뢰 ② 다소 신뢰 ③ 그저 그렇다 ④ 신뢰하지 않는 편 ⑤전혀 신뢰 않음

16. 귀하는 한국 정부 방사능 정보에 대한 신뢰는 어느 정도 입니까?

① 매우 신뢰 ② 다소 신뢰 ③ 그저 그렇다 ④ 신뢰하지 않는 편 ⑤전혀 신뢰 않음

17. 귀하는 다음과 같은 단신뉴스 보도에 대해 어떻게 생각하십니까?

| 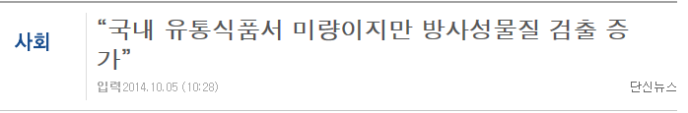 |
| --- |

| ① 매우 우려한다 | ② 우려한다 | ③ 크게 신경쓰지 않는다 |
| --- | --- | --- |
| ④ 우려하지 않는 편이다 | ⑤ 전혀 우려하지 않는다 |  |

18. 귀하는 뉴스 등 매스컴의 정보를 어느 정도 신뢰하십니까?

| ① 절대적으로 신뢰한다 | ② 다소 신뢰한다 | ③ 그저 그렇다 |
| --- | --- | --- |
| ④ 신뢰하지 않는 편이다 | ⑤ 전혀 신뢰하지 않는다 |  |

19. 귀하는 우리나라의 식품 방사능 관리는 어느 정도라고 생각하십니까?

| ① 매우 잘함 | ② 잘함 | ③ 보통 | ④ 다소 미흡 | ⑤ 매우미흡 |
| --- | --- | --- | --- | --- |

20. 귀하는 정부가 방사능관련 정보를 어떻게 제공하는 것이 가장 좋다고 생각하십니까?

| ① 정부 홈페이지 정보 게재 | ② 포털(네이버, 다음 등)에 동영상게재 |
| --- | --- |
| ③ TV매체의 기획된 프로그램 | ④ 인터넷, SNS 활용 |
| ⑤ 신문 게재 | ⑥ 자치단체 반회보 게재 |
| ⑦ 기타 ( ) |  |

21. 일본산 수산물 방사능 안전에 대해 어떻게 관리하는 것이 바람직하다고 생각하십니까?

| ① CODEX(국제식품규격위원회)의 국제기준에 준해서 |
| --- |
| ② 우리나라보다 더 엄격한 안전기준을 적용하는 국가와 똑같은 수준으로 |
| ③ 전 세계 어느 나라보다 엄격하게 |
| ④ 기타 ( ) |

| 현재 우리나라는 방사성 세슘관리를 일본과 똑같은 수준으로 관리하고 있습니다.  일본은 현재 방사능 검출량이 미량임에도 한국의 조치가 지나치다며 수입제한을 완화하거나 철회하기를 요구하고 있습니다. |
| --- |

22. 일본과의 무역마찰을 감수하더라도 현 수준과 같은 일본산 수산물 수입규제를 지속해야한다고 생각하십니까?

| ① 매우 그렇다 | ② 그렇다 | ③ 그저 그렇다 |
| --- | --- | --- |
| ④ 아니다 | ⑤ 전혀 아니다 | ⑥ 잘 모르겠다 |

23. 위 22번 문항에서 ①,②번을 선택하셨다면 그 방법은 무엇입니까?

| ① 후쿠시마 원전 방사능 문제가 해소될 때까지 일본산 식품 수입을 전면 금지해야 한다. |
| --- |
| ② 적어도 특정 지역(현)산물 또는 특정 품목(예: 수산물)에 대해서는 당분간 무조건 금지하는 것이 좋다. |
| ③ 방사능 검사결과에 따라 결정하되 기준치를 더 낮춰야 한다. |
| ④ 지금처럼 방사능을 검사하여 기준치를 넘는 것만 제한해야 한다. |

**< 일반사항 >**

| 1. 성별 | ① 남성 | ② 여성 | | |  |  | |  |
| --- | --- | --- | --- | --- | --- | --- | --- | --- |
| 2. 연령 | ① 20대 | ② 30대 | | | ③ 40대 | ④ 50대 | | ⑤ 60대 이상 |
| 3. 지역 | ① 서울 | ② 경기 | | | ③ 충청 | ④ 경상 | |  |
|  | ⑤ 전라 | ⑥ 부산 | | | ⑦ 강원 |  | |  |
| 4. 학력 | ① 고졸이하 | ② 대졸 | | | ③ 대학원 이상 | | |  |
| 5. 직업 | ① 전업주부 | ② 회사원 | | | ③ 학생 | ④ 공무원 | | ⑤ 자영업 |
|  | ⑥ 기타 |  | | |  |  | |  |
| 6. 동거가족 | ① 어린이 | | ② 초등학생 | | ③ 중학생 | ④ 고등학생 | | ⑤ 65세 이상 |
|  | ⑥ 동거인 없음 | | |  | | |  | |

수고하셨습니다.
