# Supplementary material for: Factors associated with the risk perception and purchase decisions of Fukushima-related food in South Korea
Source: PLoS One. 2017 Nov 8;12(11):e0187655. doi: 10.1371/journal.pone.0187655 (PMC5695610; doi:10.1371/journal.pone.0187655)
Supplement: S2 Appendix — (DOCX) [file pone.0187655.s002.docx]

| There has been a high level of concern by our citizens on the food products with radioactive contamination after the Fukushima nuclear power plant incident. Many problems have been revealed in Korea nuclear power plants, and the anxiety is increasing. This survey aims to identify what the citizens think about radiation and its risks to prioritize communication in the radioactive safety policies of the government, to be used as policy data.  2014.11.  Consumers Union of Korea |
| --- |

**S2 Appendix. Survey questionnaire in English**

**Ⅰ. Survey on perception research on radioactive material**

1. Have you been provided information, in form of education or other, on radiation or radioactive material?

**(Excludes news on radiation accidents)**

| ① Yes (Go to 1-1) | ② No (Go to 2) |
| --- | --- |

1-1. If you have experience being provided information, where have you received **the most** information?

| ① Mass media (TV, Newspaper, Radio) | ② Speeches and seminars | ③ School education | |
| --- | --- | --- | --- |
| ④ Internet, SNS | ⑤ Education programs from NGO | ⑥ Government webpages | |
| ⑦ Friends and acquaintances | ⑧ Flyers from government or public institutions | |  |
| ⑨ Other( ) |  |  | |

2. Please select **three** substances found in food that you feel are **very harmful.**

| Type | Harm | Type | Harm |
| --- | --- | --- | --- |
| Residual pesticide |  | GMO(Genetically modified foods) |  |
| Norovirus |  | Methylmercury |  |
| Mad cow disease |  | Food poisoning virus (coliform, O-157) |  |
| Radioactive contaminated food |  | Heavy metals (Lead) |  |
| Food additive |  | Irradiated foods |  |

3. What is the **first thing** that comes to mind when you hear the word ‘radiation’?

| ① Hospitals | ② Nuclear weaponry | ③ Nuclear power plant |
| --- | --- | --- |
| ④ Fukushima nuclear power plant incident | ⑤ Radon | ⑥ Other ( ) |

4. What is the health impact that you are the most concerned about when exposed to radiation?

| ① Cancer (incl. Leukemia) | ② Child deformity | ③ Genetic diseases |
| --- | --- | --- |
| ④ Early death from a mysterious disease | ⑤ Don’t know | ⑥ Other( ) |

5. On a daily basis, do you feel as if your health is threatened by radiation (radioactive materials)?

| ① Yes(Go to 5-1) | ② No(Go to 6) | ③ Don’t care (Go to 6) |
| --- | --- | --- |

5-1. Why did you choose ① above?

| ① Because other people think so |
| --- |
| ② Because scientists do not know well about dangers posed by radiation |
| ③ Because radiation is used daily without knowing |
| ④ Because radioactive material is continuously discharged by the Fukushima nuclear power plant or nuclear power plants in South Korea |
| ⑤ Because radiation is dangerous irrespective of amount |
| ⑥ Other ( ) |

| We can find naturally occurring radioactive material such as radon and potassium-40. Radon is natural radioactive material seeping out into air from the ground and exists anywhere. All food products contain potassium-40. While on international flights, we are exposed to cosmic radiation. The level of natural radiation exposure by our citizens is around 3.0 mSv. The iodine-131 and cesium discharged from Fukushima due to nuclear power plant incident are artificial radioactive materials. |
| --- |

| * Sievert(Sv): A measurement unit for human radiation exposure  * Becquerel (Bq): A measurement unit for radioactivity amount |
| --- |

6. Rice and Kimchi using agricultural products may have radioactivity. Which of the below types of radiation is in the **highest** concentrations?

| ① There is no radioactivity | ② Radiation from the Fukushima nuclear power plant |
| --- | --- |
| ③ Radioactive fallout from the past nuclear bomb experiments | ④ Natural radiation |
| ⑤ Radiation from Korean nuclear power plants | ⑥ Don’t know |

**II. Survey on food purchases**

7. What do you think is the most important factor in food purchases?

| ① Quality (taste) | ② freshness | ③ expiry date (period of quality maintenance) | | | ④ origin |
| --- | --- | --- | --- | --- | --- |
| ⑤ safety(residual pesticides, food additives, radioactive materials, etc.) | | | ⑥ price | ⑦ Don’t know | |

8. Which of the following products do you check origins for during purchase?

| ① All of them | ② Babyof foods | ③ Seafood |
| --- | --- | --- |
| ④ Rice | ⑤ Specific products ( ) |  |

9. What are your tendencies like for seafood purchases?

| ① Only of Korean origin |
| --- |
| ② All seafood despite of origin |
| ③ Avoid seafood from Japan only |
| ④ Do not purchase seafood despite of origin, from Korea or imported |
| ⑤ Do not purchase as telling origins apart is difficult |

10. What is your frequency of purchasing seafood after the Fukushima nuclear power plant incident?

| ① No change after incident (Go to 11) | ② Purchase frequency has decreased (Go to 10-1) |
| --- | --- |
| ③ Prchase frequency has increased (Go to 11) | ④ I do not purchase seafood (Go to 10-1) |

10-1. In Question 10, why did you choose ①, ②? Please select 2 items.

| ① Because of lingering concerns for Japanese seafood among imported seafood |
| --- |
| ② Because radioactive material is found in seafood, irrespective of amounts |
| ③ Because the information on the management of Japanese seafood cannot be trusted |
| ④ Because the markings of origins cannot be trusted |
| ⑤ Because the research results of the MFDS cannot be trusted |
| ⑥ Because the test of the MFDS is incomplete |

11. Currently, **the imported seafood from Japan** has levels of radiation that is almost non-detectable. In this case, would you purchase Japanese fish going forward?

| ① Yes, will purchase (Go to 11-2) | ② No, will not purchase (Go to 11-1) |
| --- | --- |
| ③ Don’t know (Go to 11-1) |  |

11-1. Why did you answer, “**No, will not purchase or Don’t know”?**

| ① Because the standard itself is wrong |
| --- |
| ② Because the government seems to be hiding something |
| ③ Because there may be radioactive material over the prescribed limits in the products that I bought, as not all products are checked |
| ④ Because the released radioactivity to the ocean in 2011 is back to the Pacific Ocean |

11-2. Why did you answer, “**Yes, will purchase”**?

| ① Because I think the level of non-detectability is safe enough |
| --- |
| ② Because the management by the government can be trusted |
| ③ Because it appears that Japan is doing a good job of managing the waters at issue |
| ④ Because the concerns on radioactive materials in seafood are unnecessary |

12. Did you know that the government is currently **providing information on the management of radiation on imported foods on the website of MFDS** after Fukushima nuclear power plant incident?

| ① Yes | ② No |
| --- | --- |

12-1. Do you refer to the information on harmful substances provided by MFDS when you purchase food?

| ① Yes | ② No |
| --- | --- |

**III. Research on the knowledge levels of radiation and radioactivity**

13. How well do you know the following items?(Mark it with a √a

| **Details** | **①Yes** | **②No** | **③ Donails with a** |
| --- | --- | --- | --- |
| A. There is naturally occurring radiation in the nature and all materials contain a small amount of radioactivity |  |  |  |
| B. The level of exposure to natural radiation can differ by region, house format and daily activities |  |  |  |
| C. It is same as natural radiation and artificial radiation (radiation from nuclear power plant accidents, X-ray) |  |  |  |
| D. Natural radioactivity is less harmful than artificial radioactivity |  |  |  |
| E. Iodine-131 and cesium-137 are artificial radioactive materials |  |  |  |
| F. The government has a pre-set level of acceptable radiation to the human body and actively manages exposure. |  |  |  |
| G. Unless one is exposed to radiation in large amounts during short periods of time, it is very unlikely to harm health from radiation |  |  |  |
| H. Radiation entering the body undergo natural decay or are excreted through metabolism (urine or feces) |  |  |  |
| I. Even if one were to eat fish with 100Bq (current limit) of radioactive cesium daily for a year, the level of exposure to radiation from there is same as one instance of chest x-ray. |  |  |  |

| Food radiation level in Korea is set to 100Bq/kg of cesium for all food products and managed accordingly. |
| --- |

14. What if fish had 10 Bq/kg, much lower than the standard of 100 Bq/kg?

| ① Will not purchase food with even 10 Bq/kg or 1 Bq/kg, because I do not like radiation |
| --- |
| ② While within safety standards, will not purchase as it is harmful to the body |
| ③ Will purchase as it is within safety standards |
| ④ Will feel comparatively safer compared to other products, in which I won bodyium daily for a year, the lev |

**IV. Level of confidence for radioactive material management and perceptions towards safety management policies**

16. What is your level of confidence on the **radiation information from the Japanese government**?

① Very confident ② Rather confident ③ Average ④ Rather not confident ⑤Not at all confident

17. What is your level of confidence on the **radiation information from the Korean government**?

① Very confident ② Rather confident ③ Average ④ Rather not confident ⑤Not at all confident

18. What do you think about news bulletins such as the one found below?

| Society \| Increase in detection of radioactive substances in food products distributed in Korea |
| --- |

| ① Very concerned | ② Concerned | ③ Average |
| --- | --- | --- |
| ④ Not concerned | ⑤ Not concerned at all |  |

19. What is your level of confidence on the **mass media**?

① Very confident ② Rather confident ③ Average ④ Rather not confident ⑤Not at all confident

20. What do you think of the competence of **food radiation management in Korea**?

| ① Very good | ② Good | ③ Average | ④ Rather incompetent | ⑤ Very incompetent |
| --- | --- | --- | --- | --- |

21. What do you think is the optimal form of providing **radiation-related information** by the government?

| ① Information on the government websites | ② Put films on portal websites (Naver, Daum) | |
| --- | --- | --- |
| ③ Documentaries in TVs | ④ Utilize internet or SNS | |
| ⑤ publish on newspapers | ⑥ Place it on regular mails from regional governments | |
| ⑦ Other ( ) | |  |

22. What do you think is the appropriate level of **radioactive safety for Japan-imported seafood**?

| ① In accordance to the international standards of Codex Alimentarius (CODEX) |
| --- |
| ② In accordance to the level of safety from a nation that has more stringent standards compared to Korea |
| ③ More stringent than any other nation in the world |
| ④ Other ( ) |

| Currently, Korea implements the same level of safety standards for radioactive cesium as Japan.  While the level of radiation discovered is low, Japan demands that the Korean policies are excessive and asks that we either ease the import restrictions or remove them altogether. |
| --- |

22. Do you think that even if take trade friction with Japan, should maintain **import regulations at the current levels for Japan-imported seafood**?

| ① Definitely yes | ② Yes | ③ Average |
| --- | --- | --- |
| ④ No | ⑤ Not at all | ⑥ Not sure |

22-1. If you have chosen ①,② in the above question 22, what is the method that you find fair?

| ① must ban all imports of Japanese food products until the problem of radiation from the Fukushima nuclear power plant is fully resolved |
| --- |
| ② Must ban all imports from specific region (prefecture) or on specific items (ex. Seafood) for the time being |
| ③ Decide depending on radiation tests, but lower the allowed amounts of radiation |
| ④ Limit only those in excess of allowed amounts and carry on radiation tests. |

**< General items >**

| **1. Sex** | ① Male | ② Female | | |  |  | | | |  |
| --- | --- | --- | --- | --- | --- | --- | --- | --- | --- | --- |
| **2. Age** | ① 20s | ② 30s | | | ③ 40s | ④ 50s | | | | ⑤ 60s or above |
| **3. Region** | ① Seoul | ② Gyeonggi | | | ③ Chungcheong | | | | ④ Gyeongsang | |
|  | ⑤ Jeolla | ⑥ Busan | | | ⑦ Gangwon |  | | | |  |
| **4. Education** | ① high school or below | | | ② Post-secondary | | | ③ Post-graduate school | | | |
| **5. Occupation** | ① Housewife | | | ② Professional | | | ③ student | | | |
|  | ④ public official | | | ⑤ self-employed | | | ⑥ other | | | |
| **6. Family**  **member** | ① Children | | ② elementary school | | | | | ③ middle school | | |
|  | ④ high school | | ⑤ 65 or above | | | | | ⑥ No family member | | |

Thank you.
